# Consumer satisfaction with primary care provider choice and associated trust

**DOI:** 10.1186/1472-6963-6-139

**Published:** 2006-10-23

**Authors:** Ming Ying L Chu-Weininger, Rajesh Balkrishnan

**Affiliations:** 1 School of Health Information Sciences, University of Texas Health Science Center at Houston, Houston, TX 77030, US; 2 College of Pharmacy and School of Public Health, Ohio State University, Columbus, OH 43210, US

## Abstract

**Background:**

Development of managed care, characterized by limited provider choice, is believed to undermine trust. Provider choice has been identified as strongly associated with physician trust. Stakeholders in a competitive healthcare market have competing agendas related to choice. The purpose of this study is to analyze variables associated with consumer's satisfaction that they have enough choice when selecting their primary care provider (PCP), and to analyze the importance of these variables on provider trust.

**Methods:**

A 1999 randomized national cross-sectional telephone survey conducted of United States residential households, who had a telephone, had seen a medical professional at least twice in the past two years, and aged ≥ 20 years was selected for secondary data analyses. Among 1,117 households interviewed, 564 were selected as the final sample. Subjects responded to a core set of questions related to provider trust, and a subset of questions related to trust in the insurer. A previously developed conceptual framework was adopted. Linear and logistic regressions were performed based on this framework.

**Results:**

Results affirmed 'satisfaction with amount of PCP choice' was significantly (p < .001) associated with provider trust. 'PCP's care being extremely effective' was strongly associated with 'satisfaction with amount of PCP choice' and 'provider trust'. Having sought a second opinion(s) was associated with lower trust. 'Spoke to the PCP outside the medical office,' 'satisfaction with the insurer' and 'insurer charges less if PCP within network' were all variables associated with 'satisfaction with amount of PCP choice' (all p < .05).

**Conclusion:**

This study confirmed the association of 'satisfaction with amount of PCP choice' with provider trust. Results affirmed 'enough PCP choice' was a strong predictor of provider trust. 'Second opinion on PCP' may indicate distrust in the provider. Data such as 'trust in providers in general' and 'the role of provider performance information' in choice, though import in PCP choice, were not available for analysis and should be explored in future studies. Results have implications for rethinking the relationships among consumer choice, consumer behaviors in making trade-offs in PCP choice, and the role of healthcare experiences in 'satisfaction with amount of PCP choice' or 'provider trust.'

## Background

Development of managed care in recent years has resulted in cost-containment practices such as gatekeeping, utilization review, and physician payment incentives, which are believed to undermine patient-physician trust [1-4]. Trust as a quality of healthcare measure [5-7] is important in medical treatment relationships and better health outcomes. Trust affects many important health attitudes, behaviors, and outcomes including medication adherence [8,9], therapeutic effects [10], patient-physician communication, health promotion efforts [11], disputes, likelihood of malpractice claims [12,13], and transaction costs [14].

The importance of trust and observation of declining trust [15,16] in the healthcare setting has led to the examination of its predictors. Examined variables included patient and physician characteristics and behaviors [7,16-19], quality and continuity of care [7,18,20-22], patient-physician communication [7,12,14,23,24], patient satisfaction [7,14], physician payment methods [25], gatekeeping [26] and utilization review [27]. Many managed care plans, including health maintenance organizations (HMOs), have limited provider choice [28]. Studies [7,9,20,22] have identified that the amount of physician choice is a predictor of, or was strongly associated with, provider trust. Hsu et al. found consumers reported greater trust in the provider on active physician choice, though the results became insignificant on adjustment of patient demographics [29]. However, the association of 'patient satisfaction with amount of physician choice' to 'trust in the provider' is not clear in the presence of other 'satisfaction with provider choice' variables.

Healthcare stakeholders have different agendas and conflicting goals in choice [30]. Consumers considered cost, physician choice, and coverage to be the most important criteria in choosing health plans [30-32]. Managed care plans are selective in provider contracting [33]. On the other hand, employers have applied fixed health contributions [34], flexible spending accounts, and health care reimbursement accounts [35] to contain costs. Consumers have to assume a greater responsibility in cost-sharing and more decisions in healthcare expense [36]. There is a predicament between cost, choice, and other consumer preferences. By identifying variables of 'satisfaction with amount of PCP choice', we can better understand 1) the association between physician choice and provider trust, and 2) if satisfaction with perceived amount of PCP choice was confounded by other PCP choice satisfaction variables. Findings would have implications for the enhancement of provider trust and would be of great interest to health plans, providers, or employers for the many benefits of provider trust.

The objectives of this study were to analyze domains that are predictive of provider choice satisfaction and to analyze the importance of variables that were categorized into these domains, including 'satisfaction with amount of PCP choice', on trust in the provider. A literature review driven conceptual framework that verified hypothetical domains predictive of provider choice satisfaction was adopted to identify variables for analyses [37]. The framework described five domains that may be related to PCP choice satisfaction: (1) consumer characteristics and health status, (2) information and decision, (3) trust in providers in general and trust in the insurer, (4) health plan financing and plan characteristics, and (5) provider characteristics and office management practices. These five domains each carry variables that may also be consumer preferences. Consumer preference was thus an implicit domain that overlapped with the five domains in the framework. For example, PCP being a specialist would be a provider characteristic and a consumer preference for a specialist. The five domains were abbreviated as 'consumer characteristics,' 'information and decision,' 'system and insurer trust,' 'plan characteristics,' and 'provider characteristics' respectively in this paper.

The specific aims of this study were: 1) to determine variables associated with the identified domains related to 'satisfaction with amount of PCP choice': consumer characteristics, information and decision, system and insurer trust, plan characteristics, and provider characteristics; 2) to determine the relative importance of each of these identified domains in predicting 'satisfaction with amount of PCP choice'; 3) to analyze the association between 'satisfaction with amount of PCP choice' and provider trust, after controlling for other provider choice satisfaction variables and potential confounders.

## Methods

### Design and sample

Between April and June of 1999, data about trust in the physician, in the insurer, in the medical profession, and about patient satisfaction with health care and with the insurer were collected from 1,117 adult residential households (response rate 51.4%) in the United States. Inclusion criteria were having a telephone, aged ≥ 20 years, and had seen a medical professional at least twice in the past two years [38]. The study sample was selected by random-digit dialing (exchange with replacement) and the next birthday method which enabled the random selection of an adult in households with more than one adult [39]. All participants were asked a core set of questions about trust in their regular provider, satisfaction with care, health related questions, and demographics. In addition, a random half (n = 564) were asked questions about trust in their health insurers, and the other half (n = 553) were asked questions about trust in providers in general [38]. These subsets of questions carried no overlapping observations between the two random halves. Almost all of the selected variables of this study were found in the core set of questions and a few variables were found in the subset of questions about trust in the insurer. In making trade-offs between major study variables and the sample size, the random sample (n = 564) was selected as the study sample.

The majority of the study sample (n = 564) comprised non-Hispanic whites (70.0%), female (67.0%), mean age 48.8 years (SD 17.0), who had a two or four year college degree (31.7%). In comparison, the majority of the U.S. population in July 1999 (n = 272,291,000) were Non-Hispanic white (71.9%), female (51.1%), mean age 36.4 (SD not available), and who had high school or more education (83.4%) [40,41]. In November 1999, 94.1% of the households (n = 105.4 millions) subscribed to a telephone. Hispanics and Blacks at annual income levels less than USD 20,000 were approximately three to eight percent lower in telephone subscription compared to non-Hispanic whites [42]. This study was approved by the Institute of Review Board of the University of Texas Health Science Center at Houston (Ref: HSC-SPH-04-087).

### Survey instrument and data management

Data from the random national survey have been used to develop multi-item measures of trust and satisfaction, including the Wake Forest Physician Trust Scale (WFPTS) [43], the Scale of Trust in Physicians in General [6], and the Scale of Trust in the Health Insurer [44]. The WFPTS and the Trust in the Health Insurer scale were continuous scales that measured 'provider trust' and 'trust in the insurer' respectively in this study. Provider trust has a summary score ranged 10–50 (sample mean = 41, SE 0.27). Table 1 describes the study variables selected according to the five domains of the adopted conceptual framework and their measurements. It also showed variables (in bold) that have significant association (p < .15) with 'satisfaction with amount of PCP choice' within respective domain models (see analytical methods below). The variable 'trust in providers in general' though selected for this study, could not be analyzed because the variable had no overlapping observation with other variables due to the fact that two subsets of questions were asked to two random halves of the national survey.

Study variables were examined for outliers, data distribution, frequencies, for variable recoding purpose, and to ensure that they met requirements for linear regression analyses. Variables with more than ten percent missing data were imputed. Simple independent t-tests were performed on each of these variables before imputation, to ensure no significant differences in the means of the outcome variable 'provider trust' between the groups with and without missing values [45]. The overall means of the respective variables were then used for imputation.

### Analytical methods

All descriptive and analytical statistics were conducted using the statistical software Intercooled STATA version 8.02. Variables were selected from the national dataset according to the five domains of the conceptual framework. To determine variables within each of the five domains (specific aim 1) and to determine if each domain was associated with the outcome of interest, a series of five logistic regression models (independent variables listed in Table 1), one for each domain, were used to predict the outcome 'satisfaction with amount of PCP choice', measured by the dichotomous variable 'enough PCP choice'. Variance inflation factors were used to check multicollinearity of variables within each model. Variables with an alpha of ≤ 0.15 were considered as significant for inclusion in subsequent analyses. In addition, logistic diagnostics were used to determine the issues of heteroskedasticity and model fit.

To test whether the domains with significant variables (result of specific aim 1) were predictive of 'satisfaction with amount of PCP choice' after controlling for potential confounders (specific aim 2) another set of logistic regression analyses was conducted examining the relationship between variables found significant within the 5 domains and 'satisfaction with amount of PCP choice.' An unrestricted model which included all the predictors found to be significant in analyses pertaining to specific aim 1 and confounders was compared to a restricted model that included only the significant predictor variables. This is to differentiate the effects of confounding on the relationship between significant predictor variables and 'satisfaction with amount of PCP choice'.

To analyze the association between 'satisfaction with amount of PCP choice' and provider trust, after controlling for other potential confounders (specific aim 3), multiple linear regression analyses were conducted at alpha = 0.01. 'Second opinion on the PCP' and 'years with the PCP' were included as confounding variables in the model because they were highly correlated with provider trust but were not items of the WFPTS that measured provider trust [38].

## Results

Two variables, 'income' and 'insurer trust,' involved 14% and 22% missing values respectively. Nevertheless, these two variables were rejected in the variable selection processes within domains and excluded from subsequent analyses. The mean, standard error of the mean, and standard deviation of 'insurer trust' before and after imputation were 36.5 [range possible: 11–55] (SE 0.37) SD = 7.80 and 36.6 (SE 0.29) SD = 6.89 respectively.

Table 1 shows the five models of specific aim 1 and their measurements. Variables with an asterisk indicated significant associations (p ≤ .05) within respective domains. The confounders (only related to specific aims 2 and 3) were listed in the same Table to show how they were measured. A total of 18 significant variables within four significant domains were found associated with 'satisfaction with amount of PCP choice.' The domain model of 'system and insurer trust', which had two identified variables 'trust in providers in general' and 'insurer trust', was only tested with the 'insurer trust' variable due to the dataset limitation of no overlapping observations. Coefficients of Hosmer-Lemeshow chi-square indicated good model fit of all models, except the single variable model 'system and insurer trust' and the domain was excluded from further analyses.

The 18 variables (Table 1, in bold) from the four significant domain models were: chronic health condition, visit to PCP > 10 times, speak to PCP outside medical office, main reason selected PCP (being assigned or little PCP choice), dispute with the PCP, would change PCP if could, years with the PCP, satisfaction with the PCP, satisfaction with the insurer, enough insurer choice, government insurance, insurer charged less if provider within network, insurer assigned a specific PCP, provider type, PCP always able to diagnose, PCP will listen with care and concern, PCP's care been extremely effective, and long waiting time to appointment. 'Assigned or little PCP choice' was one of the seven categories of the variable 'main reason selected PCP,' which varied in magnitude compared to the dichotomous variable, 'insurer assigned a specific PCP' in influencing the models. Variance Inflation Factor affirmed no multicollinearity concerns between these two variables and on all variables within the model.

Table 2 presents the results of logistic regression analyses examining predictors of 'satisfaction with amount of PCP choice' (i.e., the eighteen significant variables listed in Table 1) grouped by domains, after controlling for the confounders of 'satisfaction with amount of PCP choice': consumer age, gender, education, racematch, highly recommend PCP to family and friends, and high worry about health. Likelihood ratio test comparing the full and reduced models -2LL = 3.12 (p = .79) indicated the confounding variables had no significant contribution to the full model. In the reduced model, eight variables were significantly (highlighted in bold) associated with 'satisfaction with amount of PCP choice' (pseudo R^2 ^= 0.40, p < .05). Consumers who spoke to the PCP outside the medical office had 4.28 times (95% CI = 2.10, 8.73) the odds of reporting 'satisfaction with amount of PCP choice' compared to those who did not. Consumers who felt that their PCP's care had been extremely effective had 4.02 times (95% CI = 1.54, 10.54) the odds of reporting 'satisfaction with amount of PCP choice' compared to those who did not.

In addition, consumers who reported satisfaction with their insurers and who reported insurer charged less if the provider was within network had 2.54 times (95% CI = 1.45, 4.46) and 2.44 time (95% CI = 1.35, 4.41) the odds respectively of reporting 'satisfaction with amount of PCP choice.' compared to those who did not. Consumers who reported they have enough insurer choice had 2.16 times (95% CI = 1.12, 4.17) the odds of reporting 'satisfaction with amount of PCP choice' compared to those who did not. For each additional year with the PCP, consumers had 1.06 times (95% CI = 1.01, 1.12) the odds of reporting 'satisfaction with the amount of PCP choice'. On the other hand, consumers who had dispute experience(s) with the PCP had a reduced odds of 74 percent (95% CI = 0.12, 0.59) in reporting 'enough PCP choice' compared to those who did not. Consumers who experienced 'long waiting time to appointment' also had a reduced odds of 65 percent (95% CI = 0.17, 0.68) in reporting 'satisfaction with amount of PCP choice' compared to those who did not.

Table 3 presents the results of multiple linear regression analyses examining whether 'enough PCP choice' was predictive of provider trust, after controlling for the effects of the variables found to be predictive of 'satisfaction with amount of PCP choice' in the previous analyses, and after controlling for the confounders of provider trust, 'second opinion on the PCP', consumer age, gender, education, and racematch. Three observations of 'provider trust,' had very low trust scores ranged from 10 to 17. The sample mean of 'provider trust' was 41 (SE 0.27, SD = 6.07). These three outliers of 'provider trust' were non-Hispanic white females, aged between 21 and 37, have chronic health conditions, relatively low in education levels (2 attended high school or less, and 1 has some college education), and incomes towards the lower strata (2 at the range $20,000 to $39,000, and 1 below $20,000). There were no reasons to remove these three outliers. If the outliers were removed, it would influence the estimate of the regression coefficients. It would reduce the R^2 ^from 0.47 (p < .001) to 0.43 (p < .001) and would reduce the number of significant variables from eight to five in the full model. Table 3 shows the coefficient estimates of the 'provider trust' model with and without the outliers. The following describes the results without outlier removal.

The full and reduced models (n = 564) accounted for 47 percent and 37 percent of the variations in 'provider trust' (p < .001) respectively. A significant F (14, 510) = 19.68, p < .001 also indicated a linear relationship between the dependent and independent variables [45]. The Variance Inflation Factors of individual independent variables were ≤ 1.44, suggested no issue of multicollinearity among the variables. The full model showed eight significant variables associated with 'provider trust'. They were: 'enough PCP choice', 'dispute with PCP', 'years with the PCP', 'PCP's care has been extremely effective', 'long waiting time to appointment,' 'second opinion on PCP', consumer's gender being 'female', and consumer's 'education' level.

Consumers who felt that their 'PCP's care been extremely effective' was highly associated with provider trust (β = 7.1, robust SE 0.95, p < .001). The experience of having a second opinion on the PCP was negatively associated with provider trust (β = -6.44, robust SE = 0.94, p < .001). 'Enough PCP choice' has a positive association with 'provider trust' (β = 1.89, robust SE = 0.52, p < .001). 'Dispute with the PCP' and 'long waiting time to appointment' were both negatively associated with provider trust (β = -1.75, robust SE = 0.83, p < .035, and β = -1.19, robust SE = 0.56, p < .035 respectively). The consumer being female has some positive association with provider trust (β = 1.05, robust SE = 0.41, p < .01). Consumer's education level has some association with provider trust (β = 0.63, robust SE = 0.24, p < .01). 'Years with PCP' also has a slight association with provider trust (β = 0.07, robust SE = 0.03, p < .03).

## Discussions and conclusion

In this study, a total of 18 variables have been identified as associated with 'satisfaction with amount of PCP choice' when analyzed in each of the five domains of the conceptual framework. The 'system and insurer trust' domain was not fully tested due to data limitation and poor model fit. However, the variable 'trust in providers in general' of this domain had strong potential influence on interpersonal provider trust [38]. It should be reconsidered for examination in future studies. Analyses validated four of the five domains to be associated with 'satisfaction with amount of PCP choice' when each domain was examined independently. When the significant variables of these four domains were examined together in a single model to identify their relationship with 'satisfaction with amount of PCP choice', the domain 'consumer characteristics' was no longer associated with 'satisfaction with amount of PCP choice'.

Having 'enough insurer choice' was associated with 'satisfaction with amount of PCP choice' but not 'provider trust'. This appeared to be logical as plan choice preceded and necessarily impacted provider choice. Eighty-three percent of participants (n = 150) who reported enough insurer choice also reported enough PCP choice. Among participants who reported not enough insurer choice, 33 percent (n = 414) reported not enough PCP choice. 'Enough insurer choice' appeared to have some relationship with 'enough PCP choice.' 'Enough PCP choice', on the other hand, was found to be associated with provider trust. This confirmed the existing knowledge that enough or some amount of PCP choice was associated with provider trust.

When a plan had the characteristic of charging less if the providers were within network, it was strongly associated with greater odds in the reporting of 'satisfaction with amount of PCP choice.' This result suggested that this plan characteristic, which has the connotation of cost saving, was relatively popular among consumer preferences. A plan characteristic and consumer preference match explained the greater odds reported. Early studies of consumer behavior in plan choice suggested consumers were cost sensitive [46] while others saw provider choice as more important than benefits coverage [47]. Since Preferred Provider Organization (PPO) plans were gaining popularity over HMO plans in past years [48], it was not unreasonable to postulate that consumers had to make trade-offs between plan cost and provider choice, and that consumer preference might oscillate between the two depending on other factors, such as the proportion of employer contribution in health benefits.

'Years with the PCP' has some association with 'satisfaction with amount of PCP choice.' The duration of the patient-physician relationship indicated that the patient volunteered to stay with the provider for that length of time, or that they were required to stay with the provider for some reasons. Patients would have switched providers if the relationships were not good and they have a choice or a better choice. Nevertheless, involuntary provider switch behavior appeared to be more an issue of "employer-imposed disruption". Voluntary disenrollment from PCP practice was, however, associated with trust [49]. In the provider trust model, the variable 'years with the PCP' was also associated with the outcome of provider trust. In this case, the association was likely due to the issue of continuity of care, which may enhance provider trust [50].

'Long waiting time to appointment' has some negative association with both 'satisfaction with amount of PCP choice' and 'provider trust.' The consumer might not know if a certain provider has a 'long waiting time to appointment' unless they had known the provider before or had made efforts to find out how long it normally takes to secure an appointment. The variable also reflected the consumer preference for access to prompt appointments. The association with 'provider trust' would likely be due to a patient's perception of the provider's level of control in providing efficient services. "Control" refers to the physician's autonomy in providing needed services to the patient in a timely manner, which is a building block of physician trust [37].

The association of 'second opinion on the PCP' (due to concern of care) with lower provider trust involved a temporal issue of whether provider trust was already low at the time a second opinion was sought or if the second opinion confirmed the consumer's concern and hence decreased trust. This is a limitation of the cross-sectional study design not being able to measure the dynamic dimension of trust. Regardless of whether trust was low, declined before, after, or continued to decline after a second opinion on the PCP, the experience of having sought a second opinion on the PCP demarcated a decrease in trust.

Eight variables associated with 'satisfaction with amount of PCP choice' were analyzed in the model of 'provider trust' with and without potential confounders. Consumers' perception of the PCP's care being extremely effective was strongly associated with both 'satisfaction with amount of PCP choice' and with 'provider trust'. This finding was consistent with the consumer preference for professionally qualified providers [19], for having information about the provider's performance including error rates and adverse outcomes [31], and with the definitions of trust that the provider has the competencies to enhance the technical aspects of care [37].

Contrary to the existing knowledge that minorities, especially Blacks and Hispanics, preferred a provider of the same ethnic background or who speaks the same language [51], this study found that 'racematch' was not significantly associated with 'satisfaction with amount of PCP choice'. It was also interesting to find that 'consumer education' was negatively associated with 'provider trust.' It suggested the higher the education level the more likely that the consumer would "challenge" the provider's competencies in patient care. It was not clear if the high proportion of female subjects (67%) in the sample might have contributed to the association of 'consumer being female' to 'satisfaction with amount of PCP choice' and to 'provider trust'.

### Novel findings

Variables associated with 'satisfaction with amount of PCP choice' have never been explored before. This study identified eight variables that were associated with consumers' satisfaction with the amount of PCP choice. Having spoken to one's PCP outside of the medical office was strongly associated with reporting of enough PCP choice. Regardless of the contexts, interaction outside of the medical office may have created a sense of harmony with the PCP, such as having visited similar stores or lived in the same communities. Socialization out of the medical environment may also create a sense of friendship with the PCP. At the same time, the selected provider may just be the provider that the consumer preferred or knew from before. Nevertheless, interaction outside of the medical office was not associated with provider trust. This was most likely because this kind of interaction would not change the PCP's role as a healthcare agent, their autonomy and their competencies to take care of the patient.

The perception of having enough insurer choice, the consumers' report of satisfaction with their insurers, and health plans that 'charged less if providers within network' were strongly associated with 'satisfaction with amount of PCP choice'. 'Insurer satisfaction' included satisfaction with plan characteristics and/or insurer services in medical claims. Consumers who preferred cost saving would find such plan characteristic attractive and be more ready to report 'satisfaction with amount of PCP choice'. In contrast, consumers who preferred PCP choice more than cost saving would feel this plan characteristic restricted their access to preferred providers, and would be less likely to report 'satisfaction with amount of PCP choice'. Thus the associated variables appeared to reflect on the cost sensitivity of consumers [52], and not so much on the amount of PCP choice being available. In which case, even some amount of choice would be sufficient to constitute as "enough PCP choice." Results also highlighted the dilemma of trade off between increasing cost and limits in PCP choice for the consumer. On the other hand, the popularity of PPO Model over HMO plans suggested there was another sector of consumers who preferred greater access of PCP choice to cost saving [48], though it was not indicated in this study finding.

Consumers who had upset/dispute experience(s) with PCPs, or who experienced long waiting time to get an appointment, had a moderately lower likelihood of reporting 'satisfaction with amount of PCP choice'. It was not clear how serious the dispute(s) were and how frequent the dispute(s) occurred. Respondents had responded to the 'enough PCP choice' survey item before they were asked the 'dispute with PCP' item. Unless recent dispute(s) or repeated disputes made the experiences into long-term memory, the dispute experiences should not be affecting participants' response to the item of 'enough PCP choice.' Furthermore, the lower reporting of 'satisfaction with amount of PCP choice' may be an indicator that the PCP profiles were poor matches with the consumer preferences. Long waiting time to get an appointment was not associated with provider trust. Quality of and/or duration of visit may have made up for the deficiency in appointment schedules and had slight influence on provider trust, if any.

It was understood at the outset of the study that the evaluation of satisfaction with PCP choice may make a difference in responses depending on when the data was collected. It was interesting to discover that elements occurred after PCP choice or after PCP assignment may or may not "compensate" for an initial perception of the amount of PCP choice available. At the system level the implications of this study findings point to the direction of rethinking the consumerism of demanding more PCP choice. Is it necessary that consumers be given "adequate" amount of choice? Will it be better if consumers be assisted with information in selecting a PCP who best suits their preferences, or who is known to be very professional?

Furthermore, it is possible that patients may have projected their experiences with the physicians into the reporting of 'satisfaction with amount of PCP choice'. Satisfaction is the evaluation of past events. The timing of when to measure satisfaction with PCP choice is not necessarily better at one time than another. However, the interpretations of satisfaction with the amount of PCP choice measured immediately after the choice was made or given will have to be different from that measured several years after the choice was made. This study found some plan characteristics variables associated with 'satisfaction with amount of PCP choice'. The associations prompted the reconsideration of whether giving patients more PCP choice (say through health plan design) is an efficient or effective means of driving the healthcare market. The patient-physician experience may also have some influences on the perception of the adequacy in amount of PCP choice. In this study, patients had seen their PCPs at least twice in the past year, the experience with the PCP would inform the overall associations of medical encounters and thus 'satisfaction with amount of PCP choice' and 'provider trust.'

### Study strengths and limitations

A limitation of the study is that although the study sample was a random national sample, it did not match exactly the general U.S. population given the study inclusion criteria and racial differences in telephone subscription rate. The data was collected in 1999. It did not necessarily reflect all changes over the past few years in the healthcare market, such as voluntary or mandatory hospital or provider performance public reporting. Evolvement of healthcare in the past few years has included the shift towards greater consumer cost-sharing, and the popularity of PPO over HMO plans [48]. Other than the role of information on choice behavior, the dilemma of choice has been mostly on cost-choice trade-offs. The timeliness of the data for analysis should not be of concern. Despite the availability of increasing national and local publication or reports on health plan and provider performances, and information to assist plan and/or provider choice, there had been few published studies of the effects of performance data on consumer choice behavior [53]. Much about performance report utilization, information usefulness, and consumer satisfaction with information quality remained to be explored [54]. Another limitation of this study was the lack of data on performance report utilization in the national dataset which prohibited this element from being examined in any of the models.

Other limitations of this study include those inherent with secondary data analysis. The lack of overlapping observations in the variable 'trust in providers in general' prohibited its application for analyses. The cross-sectional study design did not account for the longitudinal dimensions of choice and of trust, and for the dynamic dimension of trust. Evaluation of variables of 'satisfaction with amount of PCP choice' was retrospective. For consumers who have had their PCP for many years, it was debatable whether their experiences with their PCP currently and over the years might have influenced their perceptions and hence 'satisfaction with amount of PCP choice.' As in any cross-sectional study design, results of this study can only be viewed as associations among variables with the outcomes of interest rather than causality.

### Future studies

**Figure 1:**
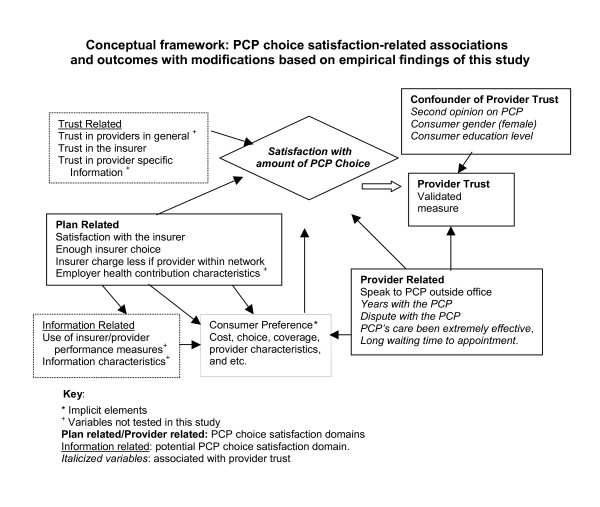
Conceptual framework: PCP choice satisfaction-related associations and outcomes with modifications based on empirical findings of this study.

Results suggested the adapted conceptual framework can be modified. Figure 1, titled 'PCP choice satisfaction-related associations and outcomes with modifications based on empirical findings of this study', illustrates the relationships of the four relevant domains. 'Consumer characteristics' was no longer significant. The revised conceptual framework constituted four renamed domains: plan related, provider related, trust related, and information related. Other than plan or provider related variables, trust or information related variables require more exploration. Conflicting information and biased marketing materials often lead to information distrust [30]. 'Trust in provider performance reports' should also be explored under the 'trust related' domain in future studies to see its influence on consumer choice behaviors.

The provider's professionalism has never been explored as a predictor of provider trust before and it deserves attention in future studies. Future studies of healthcare public reporting, of provider specific information, and of trust in information available to assist choice will unveil the roles of information in consumer preferences, in consumer satisfaction, and in provider trust. If possible, examining all the known predictors of provider trust in one study may provide a holistic view of what is relatively important in predicting provider trust and in sustaining trust.

## Abbreviations

Primary care provider (PCP)

## Competing interests

The author(s) declare that they have no competing interests.

## Authors' contributions

MYLC carried out the study including application of conceptual framework, design of the study, performance of statistical analyses, and drafting and revision of the manuscript. RB conceived of the study, participated in the design of the study, coordinated data access, critiqued the statistical methods, and helped revisions of the manuscript. All authors read and approved the final manuscript.

## Pre-publication history

The pre-publication history for this paper can be accessed here:



## Figures and Tables

**Table 1:** Variables analyzed as potential predictors of 'satisfaction with amount of PCP choice' grouped by domains

**Domain Models**	**Variables**	**Items in the National Survey**	**Var. Type §**
***Consumer Characteristics *+**	Income	< $20,000 $20,000 – 39,000 ≥ $40,000	Cat
	Chronic health condition * Poor mental health Poor physical health Visits to PCP > 10 *	Do you have a long-term medical condition? In general, would you say your mental health is excellent, good...poor (Recoded) In general, would you say your physical health is excellent, good...poor (Recoded) About how many visits have you made to (PCP) in your life? (Recoded)	Y/N Y/N Y/N Y/N
***Information and Decision ***+	Speak to PCP outside medical office *	In a typical year, about how often do you speak to (PCP) outside of the office? E.g., at the store or at a meeting (Recoded)	Y/N
	Main reason selected PCP*	What is the main reason you first selected (PCP)? Hear from family/friends Hear from other doc/person Pick from a list Assigned/no choice/only doc * Other reasons picking PCP PCP/office management related	Cat
	Dispute with PCP* Dispute with insurer Major surgery by PCP Would change PCP if could * Would change insurer if could Years with the PCP * Years with the insurer Satisfaction with the PCP* Satisfaction with the Insurer*	Have you ever been upset with/had a serious dispute with (PCP)? Have you ever had a disagreement with (insurer) e.g., over billing or coverage? Thinking back over the past 5 yrs, has (PCP) done any major surgery (Anesthesia required) for you? If you could, you would like to find a different doctor. If you could, you would like to find a different health insurer. Approximately how long has (PCP) been your doctor? About how long have you had this health insurer? Overall, you are extremely satisfied with (PCP) Overall, you are extremely satisfied with (insurer)	Y/N Y/N Y/N Y/N Y/N Cont Cont Y/N Y/N
***System & Insurer Trust***	Insurer trust	Scale of Trust in the Insurer [41]	Cont
***Plan Characteristics *+**	Enough insurer choice* Government insurer * Managed care Insurer charged less if PCP within network * Insurer assigned PCP * Permission for specialist req'd	Do you feel like you (or your spouse/domestic partner) had enough choice in selected (insurer)? Is (insurer) private insurance/government program/both private & gov't program (Recoded) What is the name of the health insurer you have used the most over the past 2 yrs? (Recoded) Does (insurer) charge you less if you choose your doctor from a list or make you pay more if you go to a doctor not on the list? Does (insurer) make you sign up with specific doctors or clinics for your routine care? Does (insurer) make you get permission before you can see a medical specialist?	Y/N Y/N Y/N Y/N Y/N Y/N
***Provider Characteristics *+**	Provider type *	What type of doctor or healthcare provider is (PCP)? (Recoded) Nurse practitioner/physician asst./unconventional healers General practitioner/internist/family doctor * Specialists	Cat
	PCP always able to diagnose *	No matter what health problem you might have, (PCP) will always be able to figure out exactly what is wrong. [including referring you to a specialist, when appropriate]	Y/N
	PCP will listen with care & Concern *	(PCP) will listen with care & concern to any problem you might have, even problems that are small or silly	Y/N
	PCP has better medical skills than most other PCPs	(PCP) has better medical skills than most other (PCP) in (his/her) field.	Y/N
	PCP's care has been extremely effective*	(PCP)'s medical care has been extremely effective	Y/N
	Long waiting time to appointment*	You have to wait too long to get an appointment to see (PCP)	Y/N
	***Confounders Tested***		
	Age Female Education:	What is your age? Interviewer record sex (Ask if necessary: Are you male or female?) (Recoded) What is the highest level of schooling you have completed? (Recoded) High school or less Vocational/technical school, some college, or 2 year college 4 year college, graduate school, or professional school	Cont Y/N Cat
	Race Match	What is (PCP)'s race? What is (participant)'s race. (Recoded)	Y/N
	Highly recommend PCP to family & friends	You would highly recommend (PCP) to your family and friends.	Y/N
	High worries about health	The healthcare you've been receiving relieves U completely of worry & uncertainty (Recoded)	Y/N
	Second opinion on PCP	Have you ever sought a 2^nd ^opinion because of concerns about the care provided by (PCP)?	Y/N

**Notes: **Outcome of each model was 'enough PCP choice' (Do you feel like you had enough choice when you selected (PCP)? Response = Yes/No).

+ Domains associated with 'satisfaction with amount of PCP choice.'

* Significant variables within domains (p < 0.15) and included in further analyses.

§ Variable Type: Cat = Categorical, Cont = Continuous

**Table 2:** Logistic regression models examining predictors of 'satisfaction with amount of PCP choice' grouped by domains (n = 564)

**Domains**	**Variables**	**Odds Ratio (SE)**		**95% C.I.**	
***Consumer***	Chronic health condition	0.932	(0.283)	0.514	1.691
***Characteristics***	Visits to PCP > 10	1.100	(0.370)	0.569	2.128
					
***Information & Decision-Making***	**Speak to PCP outside med office**	**4.276**	**(1.556)**	**2.096**	8.725*
	Reason for choosing PCP:				
	Hear from family/friends	1.522	(1.035)	0.401	5.774
	Hear from other doc/person	1.223	(0.848)	0.314	4.762
	Pick from a list	0.838	(0.617)	0.198	3.548
	Assigned/no choice/only doc	0.299	(0.218)	0.072	1.246
	Other reasons picking PCP	1.890	(1.545)	0.381	9.383
	PCP/office mgt. related	2.607	(2.325)	0.454	14.975
	**Dispute with the PCP**	**0.260**	**(0.108)**	**0.116**	0.586*
	Would change PCP if could	0.459	(0.215)	0.183	1.152
	**Years with the PCP**	**1.063**	**(0.030)**	**1.006**	1.124*
	Satisfaction with the PCP	0.931	(0.524)	0.308	2.809
	**Satisfaction with the insurer**	**2.543**	**(0.729)**	**1.450**	4.460*
					
***Plan***	**Enough insurer choice**	**2.159**	**(0.726)**	**1.117**	4.172*
***Characteristics***	Government insurer	1.735	(0.634)	0.848	3.553
	**Insurer charge less within network**	**2.441**	**(0.735)**	**1.352**	4.406*
	Insurer assigned a specific PCP	0.707	(0.210)	0.394	1.266
					
***Provider***	Provider type:				
***Characteristics***	Gen practitioner/family doc	0.388	(0.315)	0.079	1.901
	Different types of specialists	0.770	(0.631)	0.155	3.833
	PCP always able to diagnose	1.508	(0.418)	0.876	2.596
	PCP will listen with care & concern	1.731	(0.814)	0.688	4.353
	**PCP's care been extremely effective**	**4.024**	**(1.977)**	**1.536**	10.541*
	**Long waiting time to appointment**	**0.345**	**(0.119)**	**0.175**	0.680*

Notes: Outcome variable 'Satisfaction with amount of PCP Choice' was measured by the item: Do you feel like you had enough choice when you selected your PCP. Response = Yes/No.

* Significant at p < .05

**Table 3:** Linear regression models examining associations between 'satisfaction with amount of PCP choice', choice-related variables and provider trust

**Model**	**Model (n = 522) ^§^**			**Model (n = 525)**		
	Coeff.^+^	Robust SE	Non-Robust SE)	Coeff.^+^	Robust SE	Non-Robust SE
Dependent Variable	Provider Trust (Wake Forest Physician Trust Scale) Score					
						
Independent Variables						
						
**Enough PCP choice**	1.872***	**(0.530)**	**(0.535)**	1.885***	**(0.524)**	**(0.544)**
Speak to PCP outside medical office	0.839	(0.433)	(0.443)	0.126	(0.437)	(0.451)
**Dispute with the PCP**	-1.181	(0.800)	(0.727)	-1.752*	**(0.831)**	**(0.729)**
**Years with the PCP**	**0.077**^ψ^	**(0.032)**	**(0.031)**	0.071*	**(0.032)**	**(0.032)**
Satisfaction with the Insurer	0.338	(0.470)	(0.445)	0.161	(0.485)	(0.451)
Insurer charge less if provider within network	-0.132	(0.418)	(0.444)	-0.221	(0.436)	(0.428)
**PCP's care always effective**	6.740***	**(0.952)**	**(0.741)**	7.105***	**(0.953)**	**(0.750)**
**Long waiting time to appointment**	-0.886	(0.548)	(0.566)	-1.189*	**(0.563)**	**(0.572)**
**Second opinion on PCP**	-6.040***	**(0.932)**	**(0.695)**	-6.440***	**(0.940)**	**(0.701)**
Age (consumer)	-0.005	(0.011)	(0.010)	-0.000	(0.011)	(0.010)
**Female (consumer)**	1.124**	**(0.407)**	**(0.418)**	1.052**	**(0.409)**	**(0.426)**
Racematch	0.469	(0.447)	(0.447)	0.461	(0.456)	**(0.453)**
**Education**	0.569	(0.237)	(0.249)	0.632**	**(0.240)**	**(0.253)**
**Constant**	31.693***	**(1.470)**	**(1.222)**	31.24*	**(1.488)**	**(1.239)**
						
**R-squared**		**0.428**	**0.428**		**0.47**	**0.472**
**Adjusted R-squared**		**--**	**0.413**		**--**	**0.457**

***Significant at p < .001, **Significant at p< .01, *Significant at p < .035, ^ψ^Significant at p = .017

^+ ^Standardized coefficient (beta) obtained from ordinary least squares linear regression analysis.

^§ ^The model omits 3 outlier observations of the dependent variable 'provider trust'.
